# Observations on the interaction between plant growth‐promoting bacteria and the root‐knot nematode *Meloidogyne javanica*

**DOI:** 10.1002/mbo3.1319

**Published:** 2022-11-26

**Authors:** Aoife Egan, Thomais Kakouli‐Duarte

**Affiliations:** ^1^ enviroCORE, Department of Applied Science South East Technological University Carlow Ireland

**Keywords:** induced systemic resistance, *Meloidogyne javanica*, nematode development, plant biomass, plant growth‐promoting bacteria, *Pseudomonas fluorescens*

## Abstract

*Pseudomonas fluorescens*, strains L124, L228, L321, and the positive control strain F113 used in this study, produce compounds associated with plant growth promotion, biocontrol, antimicrobial and antiviral activity, and adaptation to stresses. These bacterial strains were tested in vitro and in vivo in tomato plants, to determine their potential role in *Meloidogyne javanica* suppression. In laboratory experiments, only 2% of *M. javanica* eggs hatched when exposed to the metabolites of each bacterial strain. Additionally, 100% *M. javanica* J2 mortality was recorded when nematodes were exposed to the metabolites of F113 and L228. In greenhouse experiments, *M. javanica* infected tomato plants, which were also inoculated with the bacterial strains F113 and L124, displayed the highest biomass (height, number of leaves, fresh and dry weight) of all bacterial treatments tested. Results from the development and induced systemic resistance experiments indicated that the bacterial strains F113 and L321 had the most effective biocontrol capacity over nematode infection, delayed nematode development (J3/J4, adults and galls), and reduced nematode fecundity. In addition, these results indicated that the bacterial strain L124 is an effective plant growth promoter of tomato plants. Furthermore, it was determined that the bacterial strain L321 was capable of *M. javanica* biocontrol. *P. fluorescens* F113 was effective at both increasing tomato plant biomass and *M. javanica* biocontrol. In an agricultural context, applying successional drenches with these beneficial plant growth promoting rhizobacteria would ensure bacteria viability in the rhizosphere of the plants, encourage positive plant bacterial interactions and increase biocontrol against *M. javanica*.

## Graphical abstract

*Pseudomonas fluorescens* produces compounds that increase plant growth, biocontrol, and adaptation to stress. Antagonistic effects of these strains against nematode *Meloidogyne javanica* were assessed in vitro and in vivo in tomato plants. Results suggest that *P. fluorescens* L124 is an effective plant growth promoter of tomato plants and *P. fluorescens* L321 was capable of *M. javanica* suppression. In an agricultural context, pretreating seeds and applying successional bacterial drenches would encourage positive plant bacterial interactions and increase antagonistic effects against *M. javanica*.

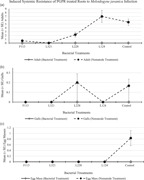

## 1 INTRODUCTION

Plant growth‐promoting rhizobacteria (PGPR), such as endophytic bacteria, can colonize living plant tissues and maintain a mutualistic relationship with the host plant. There are many benefits involved in such relationships, including increased plant growth and biocontrol against host plant pathogens (Lally et al., 2017; Otieno et al., 2015). Root colonization of these non‐pathogenic bacteria can lead to preconditioning plant defenses (Choudhary et al., 2007) by inducing host systemic resistance. That is, promoting plant health by stimulating their defense mechanisms (Romera et al., 2019) when colonized by beneficial bacterial communities. Production of secondary metabolites, mainly antibiotics, by PGPR has shown to be an efficient mechanism of nematode control (Cronin et al., 1997; Meyer et al., 2009; Siddiqui & Shaukat, 2003a). The *Pseudomonas fluorescens* strain F113, used in the present study, is a well‐researched and documented PGPR that was previously proven to be an efficient biocontrol agent against a range of plant pathogenic bacteria, fungi, and nematodes (Redondo‐Nieto et al., 2012), including the potato cyst nematode *Globodera rostochiensis* (Cronin et al., 1997). The antagonistic properties of this rhizobacterium can be attributed to the production of the antibiotic 2,4‐diacetylphloroglucinol (2,4‐DAPG), which is directly linked to the biological control of plant pathogens and the plant parasitic nematodes (PPN) *Heterodera glycines*, *Meloidogyne incognita*, and *Pratylenchus scribneri* (Meyer et al., 2009). Other strains of *Pseudomonas* are reported to produce the volatile 1H indole‐3‐carboxaldehyde, which was previously found to have a lethal effect on the bacterial feeding nematode *Caenorhabditis elegans* (Bommarius et al., 2013). In addition, it has been reported (Kim et al., 2000) that the compound benzenesulfonamide, also produced by some *Pseudomonas* species, has antifungal properties and can provide biological control of root pathogens.

Root‐knot nematodes (RKN) have a large host range and can infect more than 1700 plant species globally, resulting in major crop losses (Pulavarty et al., 2021). These types of RKN, including *Meloidogyne javanica*, are economically important PPN. They are responsible for significant crop yield losses, in particular with tomato plants, which are among the major vegetable crops grown and consumed worldwide (Perpétuo et al., 2021). In terms of controlling PPN crop infections including from RKN, currently, there are many sustainable practices and strategies employed including crop rotation, growing resistant varieties (Palomares‐Rius et al., 2021), and biological control (Pulavarty et al., 2020). Where possible, often these types of techniques are combined; however, the application of synthetic cheminematicides still dominates. Although these chemicals can be effective, they do not always kill the nematodes in the soil and do not promote sustainability. The use of such chemicals is now heavily regulated due to their hazardous effects on the environment, human health, and lack of efficacy. Plant parasitic nematodes in the soil can become resistant to these chemicals, while nematicide compounds often have nontarget effects. Therefore, the demand for improved, sustainable alternatives is increasing.

Exploiting the potential biocontrol properties of PGPR colonization in plants is a recent development considered for further investigation (Almaghrabi et al., 2013; Jiang et al., 2018; Xiang et al., 2017; Xing et al, 2020). According to dos Santos (2020), there are two systems used by PGPR to promote plant growth. The direct system involves the production of phytohormones and siderophores, nitrogen‐fixing and phosphorous solubilization, which are associated with regulating plant growth and development, reproduction, longevity, and plant death (Dilworth et al., 2017; dos Santos et al., 2020). The indirect system is associated with biocontrol by niche exclusion against phytopathogenic bacteria, thus eliciting plant systemic resistance responses. Induced systemic resistance (ISR; Choudhary et al., 2007) in plants is an enhanced state of defense that can be provoked by the presence of beneficial bacteria, increasing plant protection against subsequent biotic challenges. This enhanced state of resistance is effective against a broad range of pathogens and parasites (Choudhary et al., 2007). Plant colonization with beneficial bacteria is also effective against the development of giant cells in plant roots, preventing nematode J2 infective juveniles to establish an adequate feeding site (Martinuz et al., 2012) therefore, preventing galls from forming on the roots. Plant root colonization by beneficial bacteria also makes roots less attractive (Martinuz et al., 2012) to infective stage nematodes. If these plant defense mechanisms are triggered by a stimulus such as beneficial bacteria, before plants are infected by plant pathogens, the degree of infection can be reduced (Choudhary et al., 2007).

We hypothesized in this study, that the compounds the bacterial strains were producing would reduce nematode infection and hinder nematode development within tomato roots by eliciting an induced systemic resistance response in tomato plants. This study aimed to (1) investigate the PGPR properties of *P. fluorescens* bacterial strains in *M. javanica* infected tomato plants, (2) assess the influence of these bacteria on *M. javanica* development in tomato plants, and (3) explore the capacity of PGPR treated tomato plants to provoke an induced systemic resistance response to *M. javanica* infection.

## 2 MATERIALS AND METHODS

### 2.1 Bacterial strains and nematode extraction from roots

#### 2.1.1 Bacterial strains

*P. fluorescens* bacterial strains L124, L228, and L321 have been isolated and extensively researched at enviroCORE, in the South East Technological University, Kilkenny Road Campus, Carlow, Ireland. The *P. fluorescens* bacterial strain F113 was used as a positive control. They were used in this study to assess their capacity to promote growth in tomato plants and to assess their suppressive effects against *M. javanica*. All bacterial strains mentioned were used in every experiment in this paper.

#### 2.1.2 Nematode extraction from roots

*M. javanica* used in this study was kindly provided by Dr. Emmanuel Tzortzakakis from NAGREF, Plant Protection Institute, Heraklion, Crete, Greece. Second‐stage (J2) nematodes were extracted from tomato plant roots using a modified Baermann funnel method (Bezooijen, 2006). Infected roots were placed into a 1 mm sieve, lined with a square of loose weave muslin cloth, and the sieve was placed into a dish of sterile H_2_O for 7 days, ensuring the water was touching the roots until the J2s emerged. On emergence, they were collected on a 40 µm sieve.

#### 2.1.3 Nematode egg extraction from roots

Infected roots were placed into a screw‐topped container and vigorously agitated in 1% sodium hypochlorite solution for 3 min to release the eggs. The suspension was passed through a range of sieves from 150 to 40 µm to trap debris and soil particles in the larger sieves, and the eggs on the smaller sieves. Eggs on the 40 µm sieve were washed with sterile ddH_2_O to remove any sodium hypochlorite (Ganji et al., 2013). At this stage, the eggs were surface sterilized and utilized in the egg hatching experiment in Section 2.2. A deli dish was filled with the sterile egg suspension and incubated for 3–5 days at 28°C (Siddiqui & Shaukat, 2003a) until the sterile J2s were hatched. These nematodes were used in the ISR experiments in Section 2.3.

### 2.2 Effects of PGPR metabolites on nematode egg hatching and juvenile mortality

In total, 10 ml of a 24‐h bacterial culture (10^8^ CFU/ml) grown in nutrient broth (NB) was centrifuged twice at 2800 rpm (965 RCF) for 20 min. The supernatant was passed through a 0.22 µm sterile filter to remove the bacterial cells and tested for bacterial growth by plating 100 µl on a nutrient agar (NA) culture plate. The assay was established in sterile 24‐well tissue culture plates, with eight replications of each strain of bacterial metabolites. A suspension, 50 µl in total, containing approximately 100 surface‐sterilized nematode eggs, was added to each well holding 450 µl of bacterial supernatant. Untreated controls for the bacterial supernatant were 50 µl of nematode suspension containing surface‐sterilized eggs and 450 µl NB. The test was incubated for 5 days at 28°C (Siddiqui & Shaukat, 2003a). The wells were visually inspected for bacterial growth after 5 days of incubation. If growth was evident, the nematodes in that well were not counted. The egg hatching and juvenile mortality were then recorded.

### 2.3 Influence of PGPR on *M. javanica* development in tomato plants and on tomato plant biomass

Plastic pots, 8 cm in diameter, were filled with 350 g of sieved sterile topsoil (Westland Top Soil; a rich clay loam soil with a high humus content). In total, 25 ml of bacterial cultures (10^8^ CFU/ml) were added to the soil of each pot in the form of a bacterial drench. Bacterial cultures were utilized rather than the bacteria filtrate as in Section 2.2, as this experiment aimed to simulate natural interactions between bacteria and nematodes that would take place below ground. Subsequently, one 2‐week‐old tomato plant (*Solanum lycopersicum* var. “Moneymaker”) was planted in each pot and left to establish for 1 week before 1000 *M. javanica* J2s were added to the roots of each plant. Nematodes were added to three 22‐cm deep holes, 1 cm away from the plant base. The pots were kept on trays in a plant growth room set to 22°C with 16 h of light and 8 h of darkness. There were 18 replications of each treatment, with six plants assessed 20, 50, and 70 days after the addition of the nematodes (Martinuz et al., 2012). There were four bacterial strain treatments L124, L228, L321, and F113, and two parallel control trials established simultaneously, (1) C1 contained nematodes and no bacteria, and (2) C2 did not contain nematodes nor bacteria.

**Figure 1:**
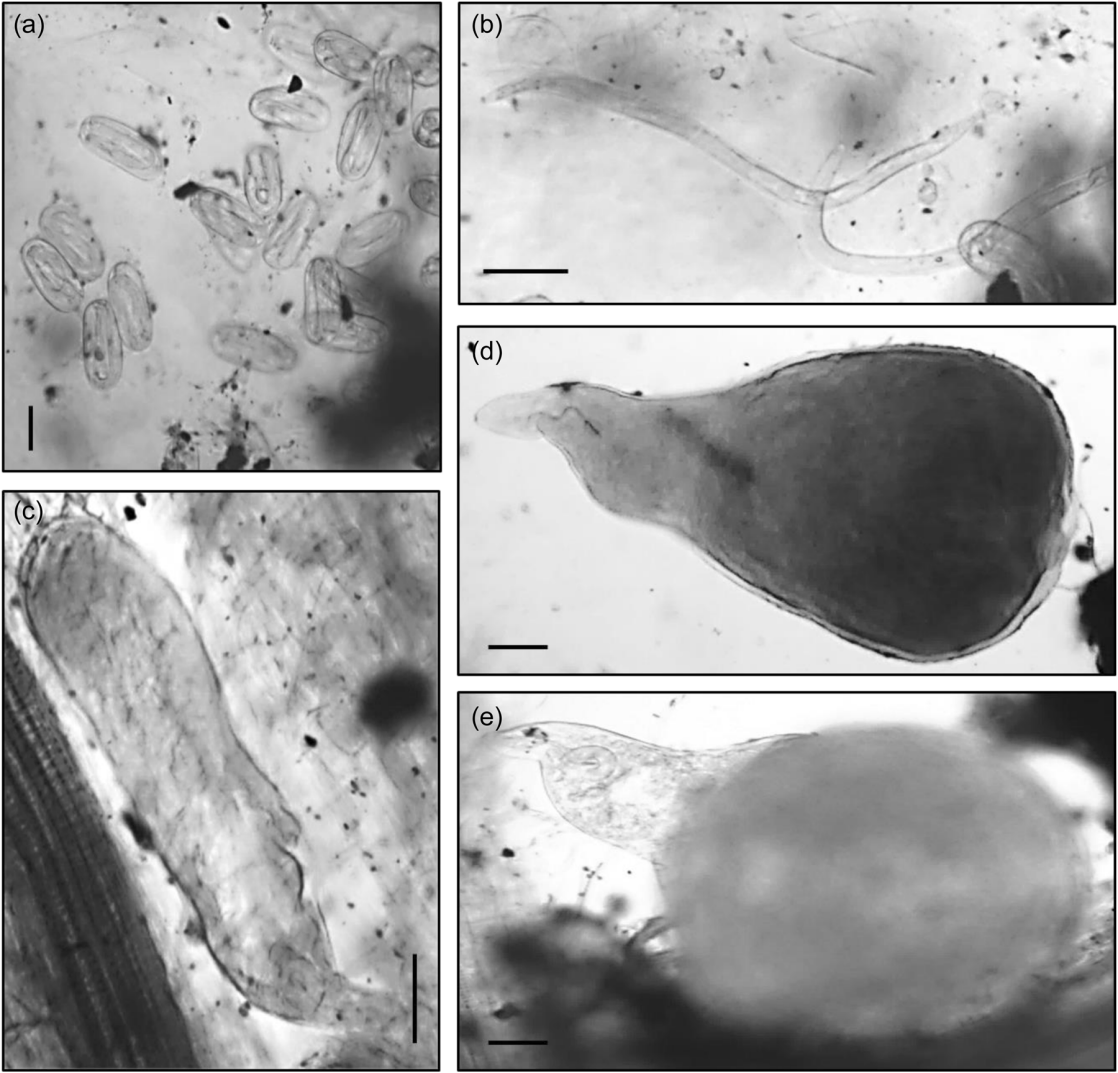
Development of *Meloidogyne javanica* from tomato plants treated with bacterial strains L124, L228, L321, and F113. Nematode developmental life stages were identified and captured with Optika Vision Pro software. (a) Eggs from an egg mass after 50 days; (b) J2 infective juvenile hatched from an egg mass after 50 days; (c) J3/J4 stage nematode inside an infection site after 50 days; (d) Young adult female located near an infection site after 20 days; Mature adult female located beside tomato roots after 50 days. Scale bars: (a) = 50 μm; (b–e) = 100 μm.

**Figure 2:**
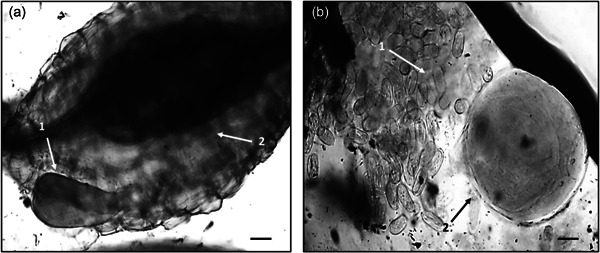
*Meloidogyne javanica* gall and egg mass on tomato plant roots treated with bacterial strains L124, L228, L321, and F113. Nematode developmental life stages were identified and captured with Optika Vision Pro software. (a1) Young adult female in a gall; (a2) Gall with enlarged, giant cells; (b1) Egg mass; (b2) Mature adult female. Scale bars: (a) = 100 μm; (b) = 50 μm.

Bacterial treatments were added to the relevant pots two additional times throughout the plant trial, after 20 and 50 days, in the form of a bacterial drench. After 20, 50, and 70 days of growth the plants were harvested, measured, weighed and the number of leaves was recorded. Plant roots were stained with acid fuchsin (Bybd et al., 1983) and mounted onto slides. The severity of nematode infection was assessed by counting the number of nematodes at different developmental stages after each harvest, using the descriptions from Dávila‐Negrón and Dickson (2013) and Eisenback and Triantaphyllou (2009) (Table 1). In addition, a high‐power microscope (Optika) was utilized to take digital images of the developmental stages (Figure 1), including the presence of galls and egg masses (Figure 2) using Optika Vision Pro software. As the nematodes were observed in the roots, their stage of development was recorded once for each treatment and the control. Once the nematode developmental stages were identified, the plant roots, stem, and leaves were oven‐dried at 60°C and the plant biomass was determined.

**Table 1:** Description of *Meloidogyne javanica* life stages for identification in tomato plant roots (Dávila‐Negrón & Dickson, 2013; Eisenback & Triantaphyllou, 2009)

Life stage	Description
J2	Vermiform shape
J3/J4s	Saccate, swollen sack‐like
Young adult female	Swollen, pear shape body, non‐egg‐laying stage
Mature adult female	Swollen, pear to globose shape body, egg‐laying stage
Galls	Cluster of enlarged plant cells or giant cells
Egg mass	Groups of eggs in a gelatinous matrix

### 2.4 Resistance of treated tomato plants to *M. javanica* infection

**Figure 3:**
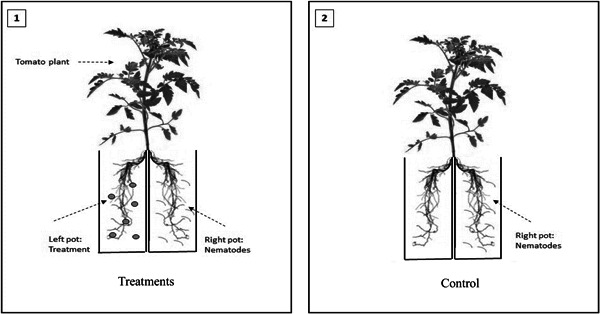
Schematic representation of the establishment of the induced systemic resistance experiment (Siddiqui & Shaukat, 2003a, 2003b, 2003b). Image 1: Bacterial strains were added to the pots on the left and *Meloidogyne javanica* J2 was added to the pots on the right; Image 2: No bacteria were added to the pots on the left but J2 was added to the pot on the right.

Surface sterilized tomato seeds (*Solanum lycopersicum* var. “Moneymaker”) were planted in pots, 8 cm in diameter, filled with 350 g of sterile soil. After 3 weeks, the tomato seedlings were uprooted and the roots were split into two halves with a sterile dissecting scalpel. Each half of the root system was immediately transplanted into one pot, 8 cm in diameter and containing 350 g of soil, and the two pots were taped together (see Figure 3). In total, 25 ml of bacterial culture was added to the root system on the left (Figure 3, Image 1). The plants were left to establish for 1 week to ensure bacterial colonization had taken place. After this time, 1000 *M. javanica* J2s were added to the root system on the right. The control treatment (Figure 3, Image 2) had nematodes added to the root system on the right with no bacterial strains added to the soil. There were six replications of each bacterial and control treatment. Thirty days post nematode infection, the plants were carefully removed from the pots, the roots were stained by boiling them in 0.1% lactic acid fuchsin (Bybd et al., 1983) and the adult nematodes, galls, and egg masses were identified in the roots and recorded.

### 2.5 Statistical analysis

Data from the nematode development, plant biomass, and ISR experiments were analyzed with the statistical package SPSS (IBM SPSS version 23). The differences among treatments were determined by a non‐parametric Kruskal–Wallis *H* test. A posthoc analysis was carried out by Dunn procedure with a Bonferroni correction for multiple comparisons to distinguish statistically significant differences among the different bacterial and the control treatments.

## 3 RESULTS

### 3.1 Effects of PGPR metabolites on *M. javanica* egg hatching and juvenile mortality

**Figure 4:**
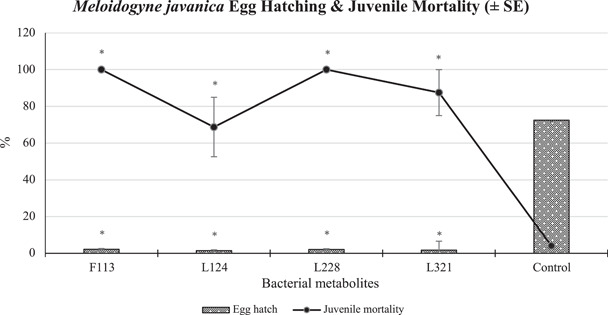
Mean (±standard error) a percentage of *Meloidogyne javanica* egg hatching and juvenile mortality when exposed to bacterial metabolites for 5 days. An asterisk (*) denotes a statistically significantly different value between the bacterial treatments and the control (*p* < 0.05).

Overall, statistically significantly (*p* < 0.001) less than 2% of PPN eggs hatched when exposed to the bacterial metabolite treatments, compared to 73% of those that hatched in the untreated control (Figure 4). *M. javanica* juveniles exposed to the bacterial metabolites of L228 and F113 in particular, suffered 100% mortality after 5 days of exposure, compared to 4% mortality in the control treatment, which was statistically significantly lower (*p* < 0.005).

### 3.2 Influence of PGPR on *M. javanica* development in tomato plants and on tomato plant biomass

#### 3.2.1 Effect of PGPR on *M. javanica* infected tomato plant growth

**Figure 5:**
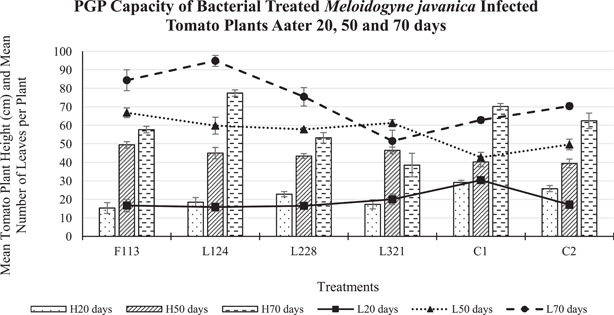
Mean (±standard error) inoculated tomato plant height and leaf number after 20, 50, and 70 days of *Meloidogyne javanica* infection. *H*: plant height; *L*: number of leaves. The error bars are ± the standard error.

Tomato plants treated with bacterial strain L228 were the tallest (23 cm) compared to those in the other bacterial treatments after 20 days (Figure 5). However, the height of all the plants treated with bacteria was shorter than those in the controls (C1 = 29 cm; C2 = 26 cm) after 20 days. After 50 days, the plants treated with bacterial strain F113 were the tallest (49 cm) of all plants including C1 (40 cm) and C2 (39 cm). The tallest plants after 70 days were those treated with L124 (77 cm), which were taller than the C1 (70 cm) and statistically significantly taller than C2 (62 cm) plants.

In addition, after 20 days, the plants treated with L321 (20 leaves) had the highest mean number of leaves, compared to the other plants treated with bacteria. However, all bacteria‐treated plants had fewer leaves compared to the control treatment C1 (30 leaves). After 50 days the plants treated with F113 had the most leaves (67 leaves) compared to these in the control treatments C1 (43 leaves) and C2 (50 leaves). Moreover, after 70 days, those treated with L124 had more leaves than all tomato plants and statistically significantly more leaves (*p* < 0.01; 95 leaves) than plants in C1 (63 leaves).

#### 3.2.2 Biomass of bacterial inoculated tomato plants infected with *M. javanica*

**Figure 6:**
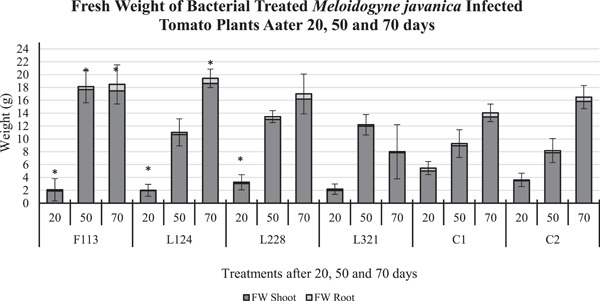
Fresh weight of bacterial‐treated tomato plants infected with *Meloidogyne javanica* after 20, 50, and 70 days. The error bars are ± standard error of the mean. FW, fresh weight. The asterisk (*) denotes a statically significant value between the bacterial treatments and the control treatments (*p* < 0.05).

The effect of the various treatments, on tomato plant fresh weight (Figure 6) after 20 days in the presence of *M. javanica* did not vary much (mean 2.5 g), with the fresh weight of plants treated with F113, L124, and L228 being statistically significantly lower than those in C1 (5 g; *p* < 0.05). However, a statistically significant increase in tomato plant fresh weight was observed in those treated with F113 (*p* < 0.05) and after 50 days (18 g), compared to C1 (9 g) and C2 (8 g). In addition, the fresh weight of those treated with L124 (19 g) and F113 (18 g) after 70 days was statistically significantly heavier (*p* < 0.05) than those in C1 plants (14 g).

**Figure 7:**
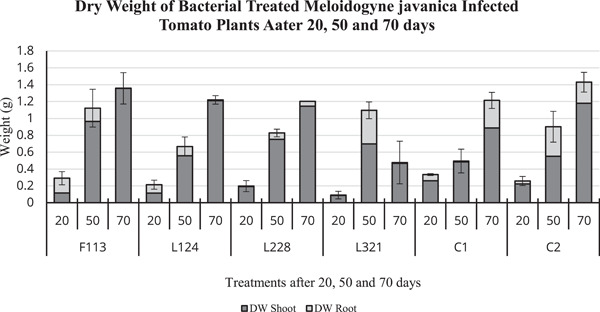
Dry weight of bacterial‐treated tomato plants infected with *Meloidogyne javanica* after 20, 50, and 70 days. The error bars are ± standard error of the mean. DW, dry weight.

On the contrary, the dry weight (Figure 7) of tomato plants after 20 days was highest in those treated with F113 (0.3 g), which was on par with C1 plants (0.3 g). After 50 days of exposure to *M. javanica*, those plants treated with F113 (1.12 g) and L321 (1.10 g) had the highest dry weight compared to C1 (0.5 g) and C2 (0.9 g). In addition, after 70 days the plants treated with F113 (1.35 g) had the highest dry weight of all treated plants, and C1, also, it was similar in weight to C2 (1.4 g).

#### 3.2.3 *M. javanica* development in tomato plants treated with PGPR strains

##### Development after 20 days

**Figure 8:**
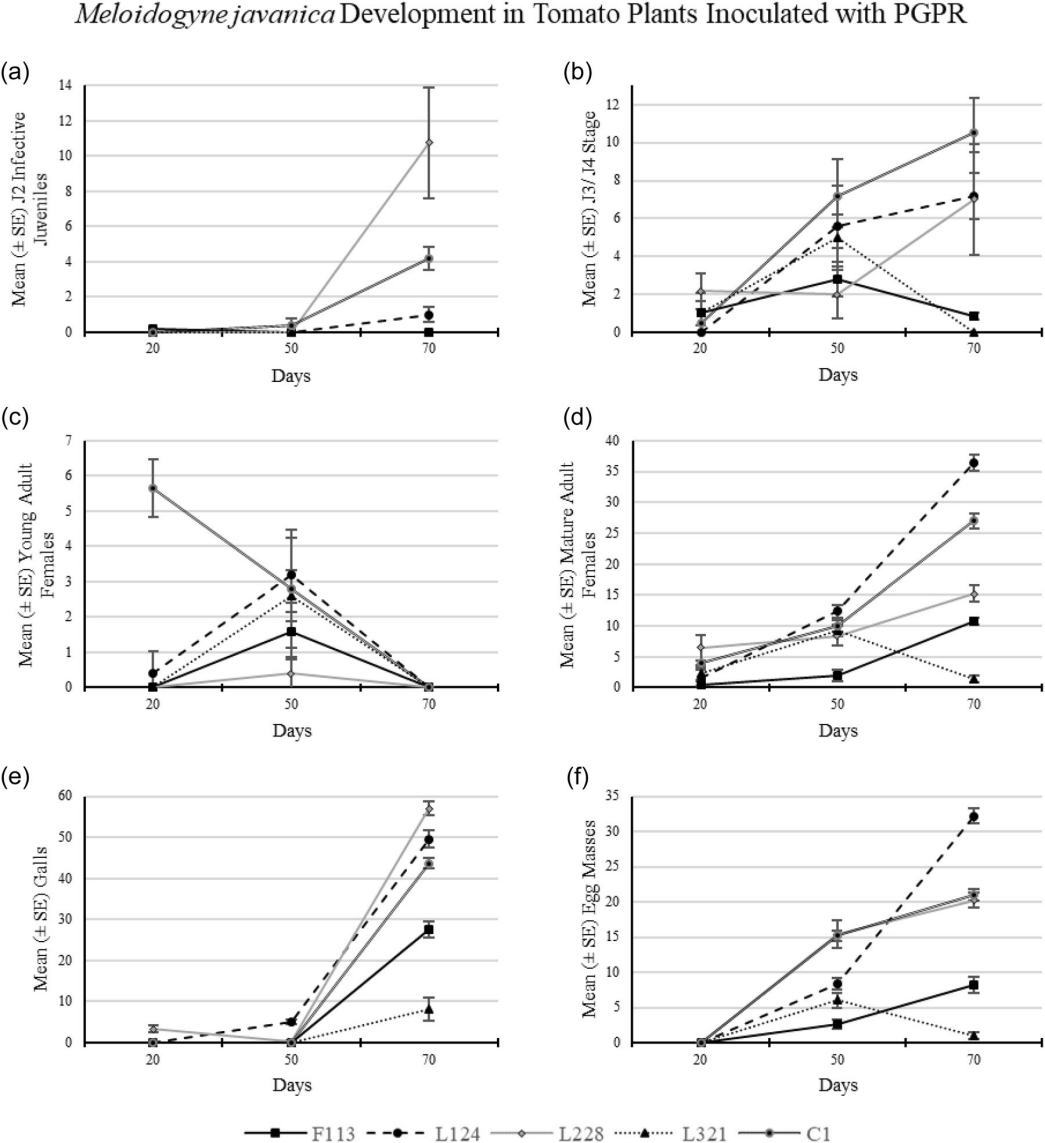
Development of *Meloidogyne javanica* in tomato plants treated with plant growth‐promoting rhizobacteria (PGPR), 20, 50, and 70 days post nematode infection. The number of (a) J2 infective juveniles, (b) J3/J4 stage, (c) young adult females, (d) mature adult females, (e) galls, and (f) egg masses observed at each time are displayed. The error bars are ± standard error.

*M. javanica* infection in tomato plants after 20 days was low, as there were no J2s (Figure 8a) present in the roots (Table 2) except in plants treated with F113 (mean 0.2 J2 per plant). The number of J3/J4 was highest in plants treated with strain L228 (Figure 8b; mean 2.1 J3/J4 per plant), compared to that in the control treatment (mean 0.5 J3/J4 per plant). In addition, there was no J3/J4 present in plants treated with L124.

**Table 2:** Presence of *Meloidogyne javanica* developmental stages that occurred in tomato plants treated with bacteria after 20, 50, and 70 days

	Time after *Meloidogyne javanica* infection		
Treatments	20 days	50 days	70 days
F113	J2	Egg mass	Galls
	Young adult female	Mature adult female	
L124	Young adult female	Egg mass	J2
	Mature adult female	J3/J4	
		Galls	
L228	J3/J4	Egg mass	J2
	Mature adult female		J3/J4
	Galls		
L321	J3/J4	Egg mass	Galls
	Mature adult female	Young adult female	
Control	J3/J4	Egg mass	All developmental stages observed
	Young adult female	J2	
	Mature adult female	Galls	

*Note*: The control treatment (C1) refers to plants infected with nematodes but were not treated with any bacterial strains.

The only plants with young adults present were those treated with L124 (Figure 8c; mean 0.4 young adults per plant), which was statistically significantly lower than the C1 plants (mean 5.6 young adults per plant). Mature adult females (Figure 8d) were found in all treated plants and those of C1. The highest number of mature adult females occurred in roots treated with L228 (mean 6.5 mature adults per plant), followed by the control treatment C1 (mean 4.0 mature adults per plant). Galls (Figure 8e) were only present in plants treated with L228 (0.33 galls per plant) at this time point. There were no egg masses present in any plants, from any of the treatments after 20 days.

##### Development after 50 days

There were no J2s (Figure 8a) observed in the roots of plants treated with bacterial treatments after 50 days, except in the control treatment C1 (mean 0.4 J2 per plant). Third and fourth juvenile (J3/J4) nematodes (Figure 8b) were found in the roots of all plants, with the highest number present in C1 (mean 7.2 J3/J4 per plant), followed by those treated with L124 (mean 5.6 J3/J4 per plant). Young adult females (Figure 8c) were found in all plants, with the highest presence in those treated with bacterial strain L124 (mean 3.2 young adults per plant) and C1 (mean 2.8 young adults per plant).

Mature adult females (Figure 8d) were found in the roots of all treatments and the highest number was present in the roots treated with L124 (mean 12.4 mature adults per plant), followed by C1 (mean 10.0 mature adults per plant). Galls (Figure 8e) were only observed in roots treated with L124 (mean 5.2 galls per plant) and L228 (mean 0.2 galls per plant), and their numbers were statistically significantly higher than those observed in the control plants. Egg masses (Figure 8f) were found in the roots of all plants, with the highest presence in those treated with bacterial strain L228 (mean 15.4 egg masses per plant) and C1 (mean 15.2 egg masses per plant).

##### Development after 70 days

The J2s (Figure 8a) were observed in tomato plant roots treated with L124 (mean 1.0 J2) and L228 (mean 10.75 J2) and in the control treatment C1 (mean 4.2 J2). J3/J4 nematodes (Figure 8b) were found in the roots of all treatments except those treated with L321. The presence of J3/J4 nematodes was highest in C1 (mean 10.5 J3/J4), followed by those in plants treated with L124 (mean 7.2 J3/J4) and L228 (mean 7.0 J3/J4). No young adult females (Figure 8c) were observed in any tomato plant roots, from any treatment after 70 days of infection.

Mature adult females (Figure 8d) were present in the roots of all treatments. However, the highest number of mature adult females was found in those plants treated with L124 (mean 36.4 mature adults), followed by C1 (mean 27.0 mature adults). Galls (Figure 8e) were present in all treatments, with the highest observed in roots treated with the bacterial strain L228 (mean 57.0 galls), followed by L124 (mean 49.6 galls) and C1 (mean 43.7 galls). Egg masses (Figure 8f) were present in the roots of all bacterial‐treated plants; however, the highest number occurred in the roots treated with L124 (mean 32.2 egg masses), followed by C1 (mean 21.0 egg masses).

### 3.3 Resistance of bacterial‐treated tomato plants to *M. javanica* infection

**Figure 9:**
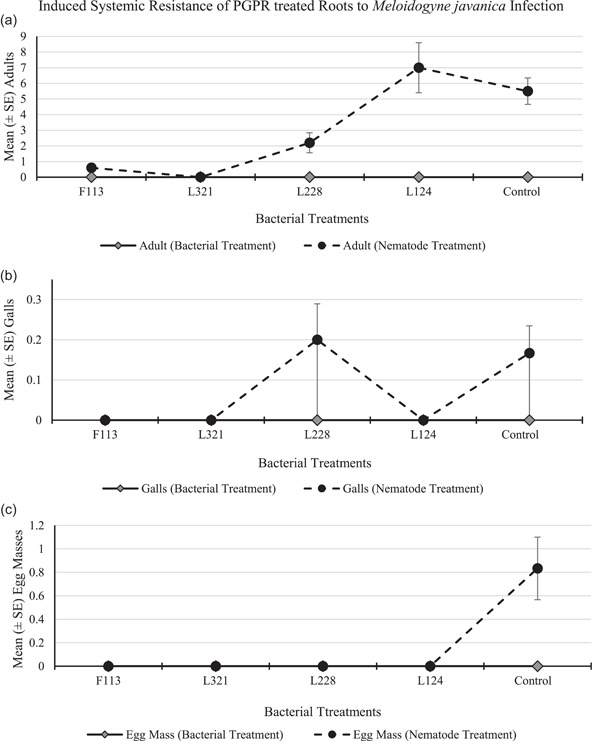
*Meloidogyne javanica* infection in tomato plants with a split root system, treated with PGPR. The mean number of (a) adult females, (b) galls, and (c) egg masses, observed in roots after 30 days. The error bars represent ± the standard error.

The root system treated with bacteria contained no nematodes, infection sites, or egg masses in any of the replications for all treatments and control (Figure 9a–c, respectively). The number of adults (Figure 9a) in the root system infected with nematodes was highest in plants treated with L124 (mean 7.0 adults per plant), followed by the control plants (mean 5.5 adults per plant). There were no galls (Figure 9b) present in the roots, except for those treated with L228 and in the control treatment (mean 0.2 galls per plant, respectively). There were no egg masses (Figure 9c) present on the roots of any of the plants for any treatment, except for the roots of the control treatment (mean 0.8 egg masses per plant).

## 4 DISCUSSION

The complex tri‐trophic interactions among *M. javanica*, beneficial bacteria, and tomato plants were explored with regard to (1) promoting the growth of *M. javanica* infected tomato plants and (2) the *M. javanica* biocontrol capability of the PGPR strains when colonizing infected tomato plants.

In a previous study (Egan, 2019), it has been determined that strain L124 produces compounds associated with biocontrol, strain L228 produces compounds linked with antimicrobial and antiviral activity and strain L321 produces compounds that are associated with adapting to stresses. In addition, *P. fluorescens* strain F113 produces the secondary metabolite 2,4‐DAPG, which has suppressive effects on a wide range of phytopathogens including oomycetes *Pythium ultimum*, the fungus *Fusarium oxysporum*, the potato‐cyst nematode *G. rostochiensis* (Redondo‐Nieto et al., 2012), and the RKN *M. incognita* (Meyer et al., 2016).

Plant growth promotion abilities of the bacterial strains were investigated in the developmental experiment by assessing plant biomass. The highest tomato plant biomass overall (including plant height, number of leaves, fresh and dry weight of stem, leaf, and roots) occurred in plants treated with bacterial strains F113 and L124. The lowest biomass occurred in tomato plants treated with bacterial strain L321, and the low effectiveness of this strain was also reported by Culhane (2016) in *Lolium perenne*. On the contrary, this was not observed by Lally et al. (2017) in *Brassica napus* or Otieno et al. (2015) in *Pisum sativum* L, who found this bacterial strain to increase biomass. This suggests that the plant root colonization and the plant growth promoting the performance of the bacterial strains are dependent on the host species.

The developmental stages of *M. javanica* were assigned according to Eisenback and Triantaphyllou (2009), who describe very little variation physically between the developmental stages J3 and J4 in *M. javanica*. They also indicate the difficulty in recognizing the differences between male and female J3 and J4 when identifying them within roots. Due to this, in the current study, any nematodes that had the appearance of a swollen version of a J2 (Figure 1) but not the characteristic globose appearance of an adult female, were classified at the J3/J4. This experiment was conducted over 70 days, much longer than previous *M. javanica* development experiments carried out (Nyczepir et al., 1999 after 24 days, and Martinuz et al., 2012 after 35 days) in tomato plants. It was important to continue this experiment for this duration to observe the plant response for longer and to gather biomass data over an extended period. In addition, it was essential to determine whether the exposure of the tomato plants to these bacteria would affect the ability of the J2s to infect the host roots or have an effect on nematode fecundity.

Overall, plants treated with the strains F113 and L321, particularly after 70 days, had the lowest number of nematodes present in their roots. In a study carried out by Martinuz et al. (2012), inoculating tomato plants with the endophytic bacterium *Rhizobium etli*, strain G12, interfered with the correct development of successful feeding sites. This obstructed or delayed J2 infection, restricting nutrient availability to the nematodes. Likewise, although different bacterial strains were utilized, in this study it was observed that there were a few J2s present in the roots up to 50 days compared to the control treatment C1. The bacterial strains used in the present study, however, could have had a similar effect on the development of feeding sites in tomato roots.

Martinuz et al. (2012) also observed that *R. etli*, strain G12, slowed the development of *M. incognita* J2s to J3 after 15 days. However, in the present study, the observed number of J3/J4 *M. javanica* was higher in the roots after 20 days compared to the control treatment C1, except in those plants treated with L124. After 50 and 70 days, the number of J3/J4 present in the roots of the treated plants was lower than those in the control plants. In addition, in those plants treated with L321 and F113, the presence of the J3/J4 decreased after 50 days. It is possible that in the present study the bacterial strains and the compounds they produce are responsible for the low numbers of J3/J4 present in the roots, in particular in the case of plants treated with L228 after 50 days and L321 after 70 days, compared to those in the control plants. The antagonistic properties of the metabolites produced by the bacteria could be responsible for this developmental delay. However, it is important to note that the lack of J3/J4 present in the roots in some instances could relate to a low number of J2s entering the roots at the initial infection stage.

It was further observed by Martinuz et al. (2012), that a reduction of adult nematodes compared to those in the untreated controls after 21 days occurred in plants treated with *R. etli*, strain G12. Similarly, the results in the present study, also show a low number of adults (except in plants treated with L228) after 20 days. Reimann et al. (2008) studied the effects of the same endophyte and nematode species as Martinuz et al. (2012) in tomato plants, but they determined that their treated plants produced fewer egg masses when compared to untreated plants after 56 days. A similar result was evident in the present study, as reduced egg masses were also observed in the 70‐day plants compared to the control treatment C1. It is clear from the results in the present study, that the treatments had a negative effect on nematode fecundity, particularly in plants treated with strains L321 and F113. The concept of priming a plant to precondition its defenses before infection (Choudhary et al., 2007) was explored in the ISR experiment in this study, using a split root system. The experiment was established to investigate possible plant resistance to nematode infection. According to Choudhary et al. (2007) and Adam et al. (2014), there have been several rhizobacterial strains found to be efficient at eliciting an ISR response in different plant species. Some of these strains, however, indicate a plant species‐specific recognition response of the receptors on the surface of root cells to the colonizing bacteria, such as in the cases of *Bacillus amyloliquefaciens* IN 937a, in *Arabidopsis*, of *Rhizobium etli* G12 in potato and *Serratia marcescens* 90‐166 in tobacco. In addition, Xing et al. (2020) state that PGPR plays an important role in plant defenses by activating ISR. They highlight that the presence of PGPR can induce a plant immune response, which can directly stimulate the plant defense responses with the production of the plant hormones salicylic acid (SA) and/or jasmonic acid (JA).

In the current study, although the bacterial strains and the nematodes were added to two separate pots, those plants treated with bacterial strains F113 and L321 were indicative of a reduction in plant infection in the pot containing nematodes, compared to the untreated pot (Figure 9). This result suggests that a systemic resistance response was induced, due to the presence of the bacterial strains on one side of the root system, which subsequently reduced infection in the roots that were exposed to the nematodes, even though they were physically located in a different pot and away from the bacteria. Similarly, Siddiqui and Shaukat (2003a) determined that *P. fluorescens*, strain CHA0, reduced *M. javanica* infection in tomato plants by eliciting a systemic resistance response in the plants, and in this case, the bacteria and nematodes were spatially separated. Likewise, Adam et al. (2014) conducted a split root ISR experiment with tomato plants, using *M. incognita* and various bacterial strains of *Bacillus subtilis* as inducers. They applied the bacteria together with the nematodes on one side of the split‐root system and found that it only slightly enhanced the antagonistic effect. In a further experiment, they found a better biocontrol effect when the nematodes and bacteria were spatially separated and therefore they deduced that ISR was identified as the major nematode control mechanism of the bacterial antagonists in their study. In addition, Xing et al. (2020) indicated that the secondary metabolites produced by PGPR, identified as ISR determinants, play a role in resisting plant pathogens by triggering the ISR response.

In the present study, further investigation on the production of SA and/or JA defense responses associated with ISR in tomato plants, using *P. fluorescens* strains against *M. javanica*, is suggested. According to Xing et al. (2020), former studies have indicated that the expression of SA and JA pathway‐related genes could be upregulated in host plants, in response to soybean cyst nematode infection and by coating seeds with bacteria. In addition, pseudomonads are known to produce a siderophore that is a precursor to the production of SA (Siddiqui & Shaukat, 2003b). Xing et al. (2020) attribute this property to *P. aeruginosa* to, improved plant resistance to *M. javanica* in addition to promoting the colonization of bacteria into the tomato rhizosphere. Therefore, it is important to understand the roles of such secondary metabolites, which can be used to inform the management of PPN.

The outcome of this study indicates that the benefits of PGPR colonization of tomato roots are two‐fold: (1) Plant biomass consistently increased at each time point, particularly in plants treated with F113 and L124 (Figures 5, 6, 7). (2) The suppressive effects of the bacterial strains against nematodes varied between the sampling times. This could be caused by early or delayed expression of the secondary metabolites produced by the bacteria. Egan (2019), found that the compound 2,4‐DAPG was expressed in the bacterial strain F113, 48 h after inoculation, but it was not produced after 5 days. On the contrary, L228 only expressed phenylacetic acid after 5 days. However, plants treated with the bacterial strains F113 and L321 consistently displayed reduced nematode infection, nematode development, and number of nematodes present in the roots, in both the development and the ISR experiment (Figures 8 and 9, respectively). Furthermore, it was evident that plants treated with the bacterial strains F113 and L321 elicited a systemic resistance response that was capable of reducing nematode infection and development within the roots.

The objective of this study was to explore the interaction between *M. javanica* infected tomato plants and bacteria and to further investigate the bacterial strains' suppressive effects against *M. javanica* and their plant growth promotion capacity. From these results, it can be recommended that there should be an emphasis on PGPR inoculation of tomato plants as early as possible for the induction of plant defense. In an agricultural context, pretreating seeds before sowing or inoculating seedlings early, before planting in the field, may be an effective approach to the management of PPNs. There is a concern, however, on the viability of the bacterial strains, in terms of their capacity to colonize host plants and the extent they would remain within the plant, to induce a systemic resistance response in a field situation (Ji et al., 2019). Application rates of the suppressive bacteria and nematode population density, in the soil and plants, play an important role in the degree of suppression of PPNs (Siddiqui & Shaukat, 2003a). Moreover, it is known that the production of antibiotics by beneficial bacterial strains, can improve their ecological fitness (Chandra & Kumar, 2017), which can further influence long‐term antagonistic effects against nematodes. Therefore, it is recommended that, along with pre‐treating seeds (Xing et al., 2020), successional drenches of PGPR on the plants or crops in an agricultural setting are important to (1) maximize the availability and viability of the bacteria in the plants, (2) encourage positive plant bacterial interactions, (3) increase plant resistance to nematode infections, and (4) facilitate bacterial biocontrol of PPN.

## 5 CONCLUSION

The results reported here suggest that some bacterial strains used in this study, and the components they produce (1) affect the capacity of *M. javanica* to infect their host and (2) delay nematode development within the roots. Through exploring the biology and behavior of these nematodes, their development in bacterially inoculated tomato plants, and the bacterial inoculated tomato plant resistance to them, two strains were identified that showed promising potential in promoting plant growth and nematode suppression. Overall, the bacterial strain L321 was the most successful for the biocontrol of *M. javanica*, and the bacterial strain L124 was the most successful strain at increasing tomato plant biomass. The positive control strain *P. fluorescens* F113 also proved to be effective at both increasing tomato plant biomass and causing an antagonistic effect against *M. javanica*.

## AUTHOR CONTRIBUTIONS

**Aoife Egan**: Data curation (lead); Formal analysis (lead); Investigation (lead); Methodology (lead); Writing – original draft (lead); Writing – review & editing (equal). **Thomais Kakouli‐Duarte**: Conceptualization (lead); Funding acquisition (lead); Methodology (supporting); Project administration (lead); Resources (lead); Supervision (lead); Writing – review & editing (equal).

## CONFLICT OF INTEREST

None declared.

## ETHICS STATEMENT

None required.

## Data Availability

All data are provided in full in this paper and are available in the Zenodo repository at https://doi.org/10.5281/zenodo.6641830
